# Chemically Modified PPARγ mRNA Unleashes Adipogenic Potential in 3T3-L1-Predipocytes: An Approach for Accelerated Wound Healing

**DOI:** 10.7150/ijms.97885

**Published:** 2024-09-23

**Authors:** Zucheng Luo, Yeheng Lu, Shaoluan Zheng, Ke Liu, Wei Fu, Yuyan Pan

**Affiliations:** 1 Department of Plastic Surgery, Zhongshan Hospital, Fudan University, Shanghai, China.; 2 Department of plastic and reconstructive surgery, Zhongshan Hospital (Xiamen), Fudan University, Xiamen, Fujian, China.; 3 Department of Dermatology, Shanghai Ninth People's Hospital, School of Medicine, Shanghai Jiao Tong University, Shanghai, China.; 4 Institute of Pediatric Translational Medicine, Shanghai Children's Medical Center, School of Medicine, Shanghai Jiao Tong University, Shanghai, China.; 5 Department of Pediatric Cardiothoracic Surgery, Shanghai Children's Medical Center, School of Medicine, Shanghai Jiao Tong University, Shanghai, China.; 6 Shanghai Key Laboratory of Tissue Engineering, Shanghai 9th People's Hospital, School of Medicine, Shanghai Jiao Tong University, Shanghai, China.

**Keywords:** PPARγ, modRNA, wound healing, adipogenic differentiation.

## Abstract

**Background:** Adipocytes play a crucial role in tissue regeneration, contributing to the restoration of damaged areas and modulating the inflammatory milieu. The modulation of gene expression through chemically modified PPARγ mRNA (PPARγ-modRNA) introduces a sophisticated approach to precisely control adipogenic processes. This study aims to explore the adipogenic potential of the PPARγ-modRNA in 3T3-L1 preadipocytes and its role in wound healing.

**Materials and Methods:** We transfected 3T3-L1 preadipocytes with PPARγ-modRNA to investigate adipogenic differentiation and cellular proliferation *in vitro*. *In vivo*, we employed a murine full-thickness skin defect model and compared the effects of modRNA-mediated PPARγ overexpression with control groups. Additionally, we conducted RNA sequencing on luciferase-modified mRNA (LUC) and PPARγ-modRNA-transfected cells (PPAR) for a comprehensive understanding of molecular mechanisms.

**Results:** PPARγ-modRNA significantly enhanced adipogenesis and proliferation in 3T3-L1 preadipocytes *in vitro*. The injection of PPARγ-modified mRNA led to accelerated wound healing compared to the control groups *in vivo*. RNA sequencing revealed upregulation of adipogenesis-related genes in the PPAR group, notably associated with the TNF signaling pathway. Subsequently, the KEGG analysis indicated that modRNA-mediated PPARγ overexpression effectively promoted adipogenesis while inhibiting TNF-α-mediated inflammation and cellular apoptosis.

**Conclusions:** This study demonstrates the innovative use of PPARγ-modRNA to induce adipogenesis and expedite wound healing. The nuclear expression of PPARγ through modRNA technology signifies a notable advancement, with implications for future therapeutic strategies targeting adipogenic processes and the inhibition of inflammation in the context of wound healing.

## Introduction

Wound healing is a complex and dynamic process that involves the coordinated interplay of various cellular and molecular events aiming to restore tissue integrity and functionality^1^, ^2^. The process of wound healing can be broadly categorized into three overlapping phases: inflammation, tissue formation, and tissue remodeling^3^. The initial inflammatory phase is crucial for clearing debris, pathogens, and damaged cells from the wound site^4^. Remarkably, adipose tissue, traditionally recognized for its role in energy storage, has emerged as a key player in the wound healing cascade^5^. Specifically, adipogenic differentiation contributes significantly to the modulation of inflammation, angiogenesis, and tissue regeneration in the wound microenvironment^6^. Furthermore, adipose tissue acts as a rich source of growth factors, cytokines, and extracellular matrix components, thereby exerting profound influences on reparative processes during wound healing^7^. Despite the intrinsic reparative capabilities of the human body, conditions such as diabetes, vascular insufficiency, and immunological disorders can disrupt the delicate balance of the wound healing cascade, leading to chronic wounds and delayed recovery^8^. Exploring innovative strategies becomes crucial in these clinical scenarios to overcome the difficulties posed by underlying medical conditions. Enhancing the regenerative potential of tissues involves addressing specific challenges related to cell migration, proliferation, extracellular matrix formation, and immune modulation in the context of impaired healing^9^.

Kindlin-2, a crucial regulator of cellular processes such as cell adhesion, migration, and signaling, has been emerged as a promising factor in the context of wound healing^10^. Our previous study has revealed that kindlin-2 exhibits a remarkable capability to enhance 3T3-L1 adipocyte differentiation and expedite wound closure, with findings indicating that its promotion of wound healing is mediated through the activation of the PI3K/AKT/mTOR signaling pathway^11^. This activation, in turn, facilitates the expression of key adipogenic genes, including PPARγ. This leads to an intriguing question whether it is possible to promote PPARγ expression, bypassing the need for an upstream signaling cascade, and potentially improve adipogenic-related outcomes. In other words, understanding whether direct modulation of PPARγ expression, without the involvement of a series of upstream signaling events, holds the potential to streamline the adipogenic differentiation process and enhance its effectiveness. Exploring the direct impact of PPARγ expression could unveil a more direct and efficient pathway for promoting adipogenesis, shedding light on novel mechanisms that might simplify the regulatory network involved in wound healing. This insight could have significant implications for developing targeted therapeutic interventions, potentially optimizing the efficiency of adipogenic differentiation for enhanced wound repair.

Recent advancements in molecular biology and RNA-based technologies have provided exciting opportunities to modulate cellular processes with unprecedented precision. The field of wound healing has witnessed a paradigm shift with the advent of RNA-based technologies, particularly the use of chemically modified mRNA (modRNA)^12^, ^13^. modRNA offers a versatile platform for precise and transient gene expression modulation, presenting a promising avenue for therapeutic applications in tissue repair^14^, ^15^. Unlike traditional gene therapy approaches, modRNA allows for controlled and temporary expression of specific genes, alleviating concerns associated with long-term genetic modifications. Our prior investigations explored the therapeutic potential of modRNA in diverse contexts, including skin flap repair and management of cutaneous defects^12^, ^16^. However, whether modRNA can effectively overexpress transcription factors and drive nuclear effects remains unanswered.

Peroxisome proliferator-activated receptor γ (PPARγ), is a nuclear receptor belonging to the PPARs family and plays a crucial role in regulating physiological processes such as lipid metabolism, glucose metabolism, and inflammatory responses^17^. PPARγ is involved in overall energy balance, adipocyte formation (adipogenesis), immune regulation, and vascular genesis, playing a pivotal role in cellular processes relevant to wound healing^18^. Its involvement in adipogenic differentiation and anti-inflammatory responses positions it as a potential therapeutic target for enhancing tissue regeneration^19^, ^20^.

Building on this foundation, in this study, our primary objective is to interpret the effects of PPARγ-modRNA on adipogenic differentiation and wound healing. Kindlin-2 serves as a positive control, validating our experimental approach and demonstrating the specific contribution of PPARγ modulation. This study explores the application of modRNA in the context of wound healing, with a specific focus on enhancing the expression of PPARγ, a key transcription factor associated with adipogenesis. This investigation represents a pioneering effort to extend the application of modRNA beyond cytoplasmic expression, targeting the nucleus to overexpress PPARγ. In this manner, we aim to unravel the molecular mechanisms underlying the potential benefits of PPARγ modulation in adipogenic differentiation and wound healing. The outcomes of this study hold the promise of advancing our understanding of modRNA technology, presenting new avenues for therapeutic interventions in the complex processes of tissue repair and regeneration.

## Materials and Methods

### modRNA synthesis and formulation

The mRNA was synthesized *in vitro* following established procedures^21^, transcribed using T7 RNA polymerase from a linearized DNA template featuring generic 5'- and 3'-UTRs, and poly-A tails. Purification of the RNA was carried out using Ambion MEGA clear spin columns. Subsequently, Antarctic Phosphatase (New England Biolabs) was applied for a 30-minute incubation at 37°C to eliminate residual 5'-phosphates. Spectrophotometers from Thermo Scientific were employed to assess the purity and concentration of the RNA. For optimal use, the purified RNA was resuspended at a concentration of 1 µg/µl in a solution containing 10 mM Tris HCl and 1 mM EDTA. In particular, N1-methylpseudouridine was employed to replace uridine in the mRNA. The sequences for luciferase were employed as per previous descriptions^22^. The sequence of the PPARγ modRNA was designed based on the NCBI Reference Sequence: NM_011146.4.

### Cell culture, transfection, and differentiation *in vitro*

3T3-L1 preadipocytes were obtained from the Chinese Academy of Sciences' cell bank in Shanghai, China, and cultured in Dulbecco's Modified Eagle Medium (DMEM, high-glucose, HyClone, USA) supplemented with 10% fetal bovine serum (FBS, Invitrogen, USA). The transfection of luciferase and PPARγ modRNA into 3T3-L1 preadipocytes was conducted using the Lipofectamine Messenger MAXTM Reagent (Invitrogen, Life Technologies, USA), following a well-established protocol^16^. In a concise procedure, 3T3-L1 cells were cultured in 6-well plates at a density of 2 × 10^5^ cells/well for 24 hours at 37°C in a humidified atmosphere containing 5% CO_2_. Subsequently, the cells were exposed to fresh reduced-serum medium Opti-MEM (Gibco, Life Technologies, USA). Specifically, 2 μl of modRNA (1 μg/μl) was mixed with 98 μl Opti-MEM in tube A and incubated for 5 minutes. Simultaneously, 5 μl of Lipofectamine was mixed with 95 μl Opti-MEM in tube B and incubated for 5 minutes. Successively, tubes A and B were combined and incubated for 20 minutes at room temperature. The medium was then aspirated, and the cells were washed twice with phosphate-buffered saline (PBS) before adding 1 ml fresh Opti-MEM to each well. To induce adipogenic differentiation, cells were incubated in an adipogenic differentiation medium kit (Cyagen, China). Following a 21-day incubation in accordance with the provided protocol, the cells were fixed using a 4% paraformaldehyde solution for 30 minutes. Subsequently, they were stained with Oil Red O (Cyagen, China) for an additional 30 minutes at room temperature.

### Cell treatment

For the control (CON) group, cells were treated with PBS. The kindlin-2 (KIN) group involved the use of 3T3-L1 stable transgene strains overexpressing kindlin-2 based on our previous study^11^. In the PPAR group, a mixture containing 2 μg modRNA was added to each well. The LUC group received an equivalent volume of luciferase modRNA. After an overnight transfection, the medium was replaced with DMEM, and cells were cultured under standard conditions. For the PPAR+TNF and LUC+TNF groups, cells in the LUC and PPAR groups were exposed to 5 ng/mL TNF-α for 24 hours, as per a previous study^23^.

### Wound healing assay

Cells were first seeded into six-well plates, and uniform scratches were meticulously created in the cell layer for each experimental group using a 200-μl pipette tip. Microscopic images of the wounds were captured immediately after scratching and at subsequent time points. These images were then used for calculating the migrated area in each well.

### RNA-Sequencing

RNA was extracted from three independent biological replicates and assessed for quality using a 2100 Expert Bioanalyzer from Agilent. Following this, library preparation and sequencing were performed on the Illumina Hiseq2000 platform at Majorbio Biotech (Shanghai, China). The subsequent data analysis was carried out using the Majorbio I-Sanger Cloud Platform (www.i-sanger.com).

### Transwell migration assay

Cells (1×10^4^ cells/well) were seeded in the upper chamber of a transwell chamber (Corning, USA) containing serum-free medium, while the lower chamber was filled with 500 μl of DMEM supplemented with 10% FBS. After a 24-hour incubation, the cells that migrated were fixed and stained using a solution of 4% paraformaldehyde and 0.4% crystal violet. Subsequently, an evaluation of the migrated 3T3-L1 cell was performed.

### Enzyme-linked immunosorbent assay

Following the manufacturer's instructions, enzyme-linked immunosorbent assay (ELISA) kits from Research and Diagnostic (R&D) Systems, MN, USA, were employed. The ELISA analysis targeted the measurement of TNF-α levels in the skin wounds of each experimental group at various time points post-surgery.

### Western Blot

Protein extraction was performed using RIPA buffer supplemented with 1 mM PMSF (EpiZyme, China). The extracted proteins were loaded onto an SDS-PAGE gel, subsequently transferred onto PVDF membranes (Millipore, USA), and blocked using QuickBlock blocking buffer (Beyotime, China). Primary antibodies (PPARγ, CCAAT/enhancer-binding protein alpha (C/EBPα), Adiponectin, Fatty Acid-Binding Protein 4 (FABP4), Tumor necrosis factor receptor-associated factor 5 (TRAF5), IL-6, Mixed lineage kinase domain-like protein (MLKL), Caspase-2, BCL-2, and GAPDH from Proteintech, China) were incubated overnight at 4 °C. Following this, HRP-conjugated secondary antibodies (Proteintech) were applied for 1 hour at room temperature. The visualization of protein bands was achieved using an ECL Luminescence Reagent Kit (EpiZyme), and subsequent analysis was conducted with ImageJ software.

### Real-time PCR

Total RNA extraction process used the EZ-press RNA Purification Kit (ZScience Biotechnology Corporation, USA), followed by reverse transcription into cDNA using the PrimeScript RT Master Mix (Takara, China). Amplification monitoring was conducted with SYBR Green (Yeasen Biotechnology, China) on the QuantStudio system (Thermo Fisher Scientific, USA). GAPDH served as the internal control, and differential expression was computed through the 2^-ΔΔCt^ method. Detailed primer information for real-time PCR analysis is outlined as follow:

GAPDH-F: AAC GAC CCC TTC ATT GAC; GAPDH-R: TCC ACG ACA TAC TCA GCA C;

PPARγ-F: GGA ATC AGC TCT GTG GAC CT; PPARγ-R: GTG GAG CAG AAA TGC TGG AG;

### Animals model

C57BL/6 mice aged 7-8 weeks were sourced from the Chinese Academy of Sciences' Animal Center in Shanghai, China. With established protocols, a circular dorsal skin wound with a 6 mm diameter was created by excising the entire skin beneath the dorsal fascia, providing a standardized wound defect model^12^. Immediate images of the wounds were captured using a digital camera. To ensure randomness and fairness, mice were then randomly assigned to 4 distinct treatment groups: the control group (Con group), mice treated with luciferase modRNA-transfected 3T3L1 cells (LUC group), those with stable transgene strain overexpressing Kindlin-2 (KIN group), and mice treated with PPARγ-modRNA-transfected 3T3-L1 cells (PPAR group). The treatment involves immediate postoperative subcutaneous injections at 3, 6, 9, and 12 o'clock around the wound edge, with 0.02ml administered at each point, with a cell density of 2×10^6^/cm^2^ based on our previously study^16^. The extent of wound healing was quantified by tracing the wound margins and calculating the percentage area relative to the original wound, using ImageJ software (NIH, Bethesda, MD, United States). Throughout the study, each mouse had ad libitum access to water and food in its cage. The experimental protocol was conducted in strict accordance with the guidelines of the Animal Care Committee of the hospital (Approval No.2024-162).

### Immunohistochemistry

Tissue specimens were paraffin-embedded and sectioned into 4 µm thick slices. After deparaffinization, rehydration, and antigen retrieval, sections were subjected to an overnight incubation with primary antibodies (PPARγ, C/EBPα, Adiponectin, and Ki67 from Proteintech, diluted at 1:100) at 4 °C. Subsequently, a 30-minute incubation with an HRP-labeled secondary antibody was performed at room temperature. The sections were then treated with DAB reagent for 10 minutes and counterstained with hematoxylin (HE). For a comprehensive histological examination, additional HE staining was conducted. Masson staining was employed to evaluate collagen deposition. The resulting images were captured using an Olympus microscope.

### Immunofluorescence

Cells were directly prepared for immunofluorescence after washing, while tissue specimens underwent paraffin embedding and were sectioned into 4 µm-thick slices. The tissue sections were then subjected to deparaffinization, rehydration, and antigen retrieval. Subsequently, they were incubated overnight at 4 °C with primary antibodies (PPARγ, C/EBPα, FAPB4, Adiponectin, and Ki67 from Proteintech, diluted at 1:100). Following the primary antibody incubation, a fluorescently labeled secondary antibody was applied to both cells and tissue sections. Imaging was performed using an Olympus fluorescence microscope for both cell cultures and tissue specimens.

### Statistical analysis

The results are presented as mean ± standard deviation. Statistical significance was assessed using GraphPad with a one-sided Student t-test or ANOVA, as deemed appropriate. The collected data demonstrated a normal distribution with similar variances, and the analysis assumed equal variances.

## Results

### PPARγ-modRNA construction and expression enhance cellular proliferation and migration in 3T3-L1 preadipocytes

**Figure 1:**
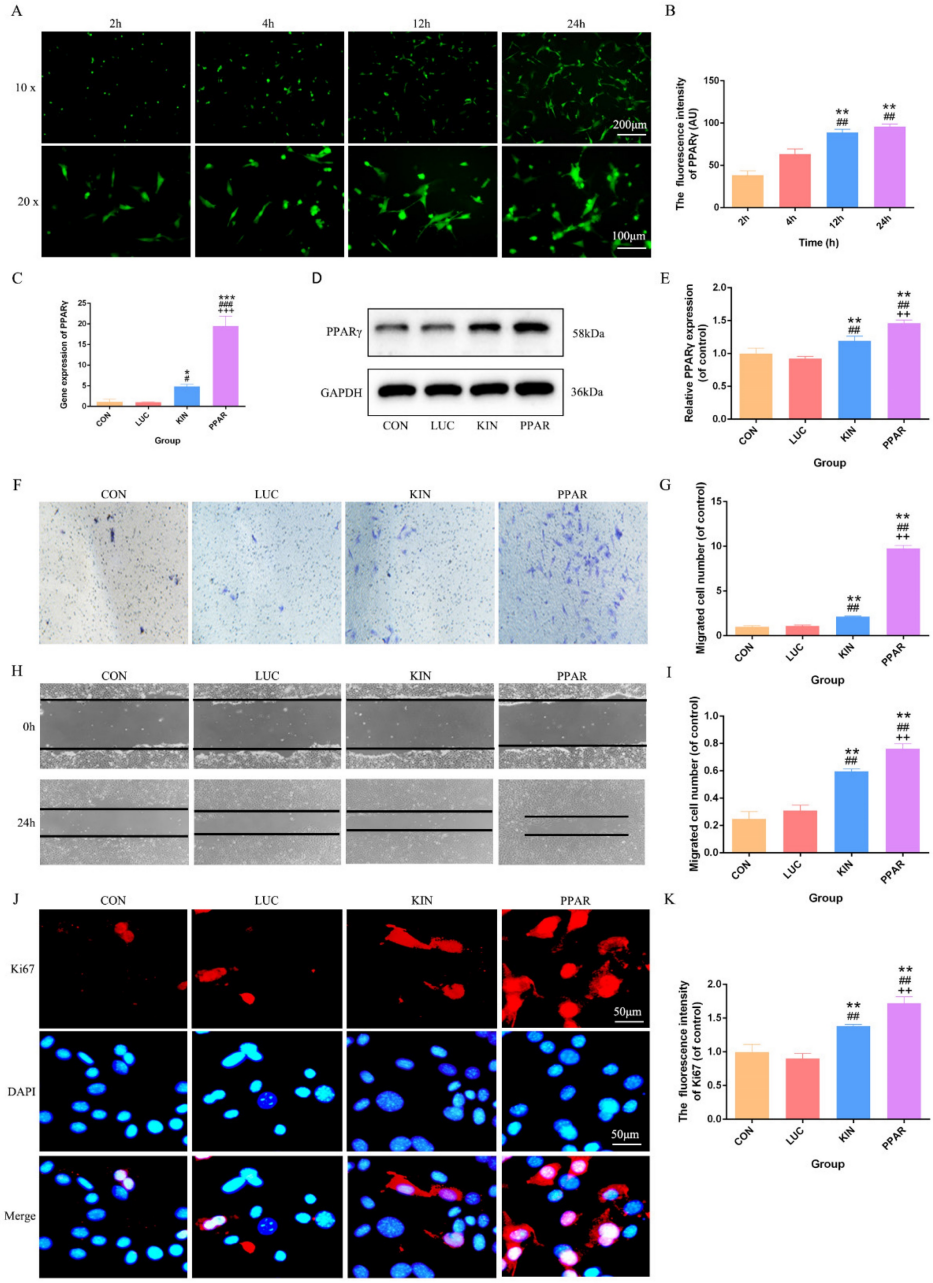
** PPARγ-modRNA transfection and functional assessments in 3T3-L1 preadipocytes.** Immunofluorescence staining at various time points (2, 4, 12, and 24 hours) post-transfection demonstrates a time-dependent increase in PPARγ fluorescence intensity (A-B). PCR and Western Blot analyses reveal the expression levels of PPARγ after 24 hours across different treatment groups. The PPAR group exhibits the highest PPARγ expression, followed by the KIN group, while the CON and LUC groups show comparable levels (C-E). Transwell and cell scratch assays demonstrate the enhanced proliferation and migration capabilities of 3T3-L1 preadipocytes. in the PPAR group compared to the KIN group (F, H). Statistical significance underscores the notable improvement in both aspects (G, I). Ki67 staining validates increased proliferation potential in the PPAR group (J-K). Statistical significance is indicated by *p < 0.05, **p < 0.01, ***p < 0.001, vs CON; #p < 0.05, ##p < 0.01, ###p < 0.001, vs LUC; +p < 0.05, ++p < 0.01, ++p < 0.001, vs KIN.

Upon successful construction of PPARγ modRNA, we initiated the transfection of 3T3-L1 preadipocytes. Immunofluorescence staining at various time points (2, 4, 12, and 24 hours) revealed a time-dependent increase in PPARγ fluorescence intensity. This observation aligned seamlessly with our prior studies, establishing a progressive and successful expression of PPARγ in the transfected cells (Fig. 1A-B). Based on this, we quantified PPARγ expression after 24 hours across different treatment groups. PCR and Western Blot analyses consistently showcased the highest PPARγ expression in the PPAR group, followed by the KIN group, while the CON and LUC groups exhibited comparable levels (Fig. 1C-E). With the confirmed PPARγ expression in hand, we proceeded to conduct functional assessments. Transwell and cell scratch assays were employed to gauge the proliferation and migration capabilities of 3T3-L1 preadipocytes. Strikingly, the PPAR group exhibited a remarkable enhancement in both aspects compared to the KIN group, with statistical significance observed (Fig. 1F-I). These findings were further validated by Ki67 immunofluorescence staining, which emphasized an increased proliferation potential in the PPAR group (Fig. 1J-K). In summary, our method, from PPARγ-modRNA construction to functional assessments, confirms success and highlights its potential therapeutic impact on wound healing, emphasizing experimental coherence and significance.

### Adipogenic differentiation validation in 3T3-L1 preadipocytes transfected with PPARγ-modRNA

**Figure 2:**
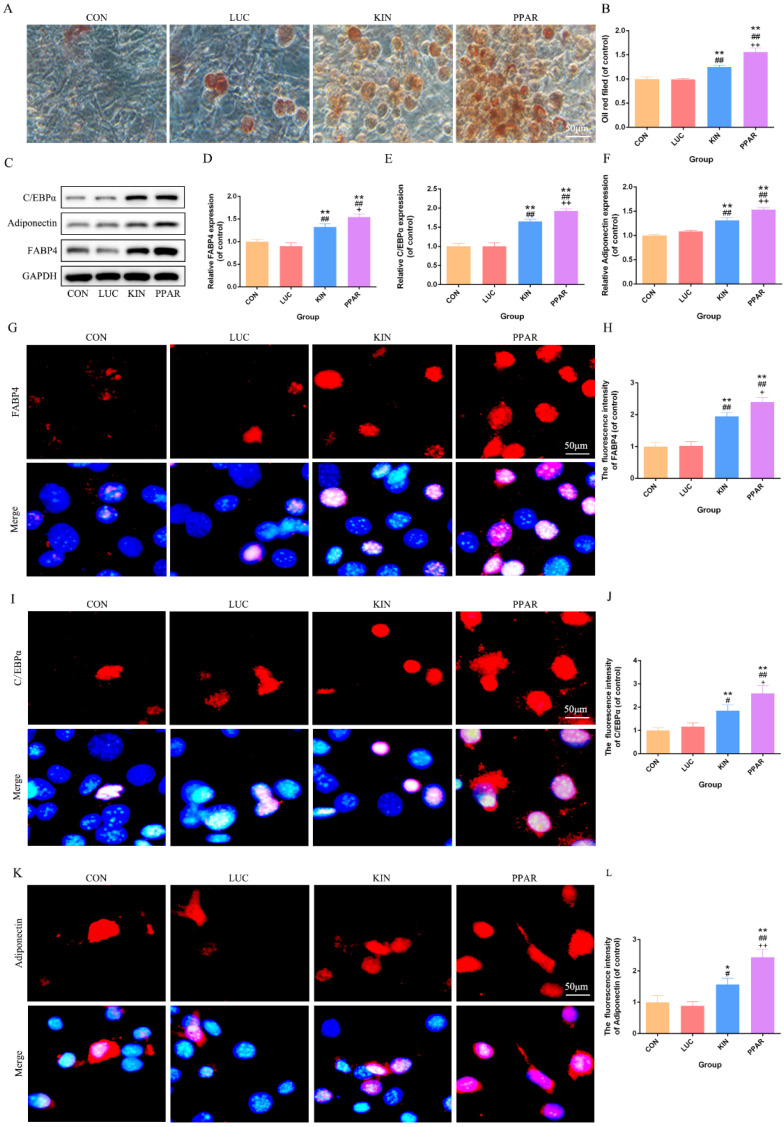
** Adipogenic Differentiation Assessment in 3T3-L1 Preadipocytes.** Transfected with PPARγ-modRNA. Following 21 days of adipogenic differentiation, Oil Red O staining reveals significantly higher lipid accumulation in the PPAR group compared to the KIN, LUC, and CON groups (A). Statistical significance is notably observed in the comparison between the KIN group and both the LUC and CON groups (B). Western Blot analysis demonstrates a marked increase in the expression of adipogenic differentiation-associated proteins, including C/EBPα, Adiponectin, and FABP4, in the PPAR group (C-F). Immunofluorescence results depict intensified fluorescence intensity for C/EBPα, Adiponectin, and FABP4 in the PPAR group (G-L). Statistical significance is indicated by *p < 0.05, **p < 0.01, ***p < 0.001, vs CON; #p < 0.05, ##p < 0.01, ###p < 0.001, vs LUC; +p < 0.05, ++p < 0.01, ++p < 0.001, vs KIN.

Following the induction of adipogenic differentiation for 21 days, Oil Red O staining revealed a significantly higher lipid accumulation in the PPAR group compared to the KIN, LUC, and CON groups (Fig. 2A). The statistical significance was particularly evident in the comparison between the PPAR group, which exhibited the highest expression, and both the LUC, KIN, and CON groups (Fig. 2B). Western Blot analysis further supported these observations, indicating a marked increase in the expression of adipogenic differentiation-associated proteins, such as C/EBPα, Adiponectin, and FABP4, in the PPAR group (Fig. 2C-F). Immunofluorescence results reflected these findings, with C/EBPα, Adiponectin, and FABP4 exhibiting more pronounced fluorescence intensity in the PPAR group (Fig. 2G-L). This trend aligned consistently with the Western Blot results, reaffirming the heightened adipogenic differentiation capacity in the PPAR group compared to the CON and KIN groups. These results indicated a robust and effective enhancement of adipogenic differentiation in 3T3-L1 preadipocytes transfected with PPARγ-modRNA, emphasizing the potential therapeutic impact of this approach in promoting adipogenesis.

### Validation of PPARγ-modRNA application in wound healing

**Figure 3:**
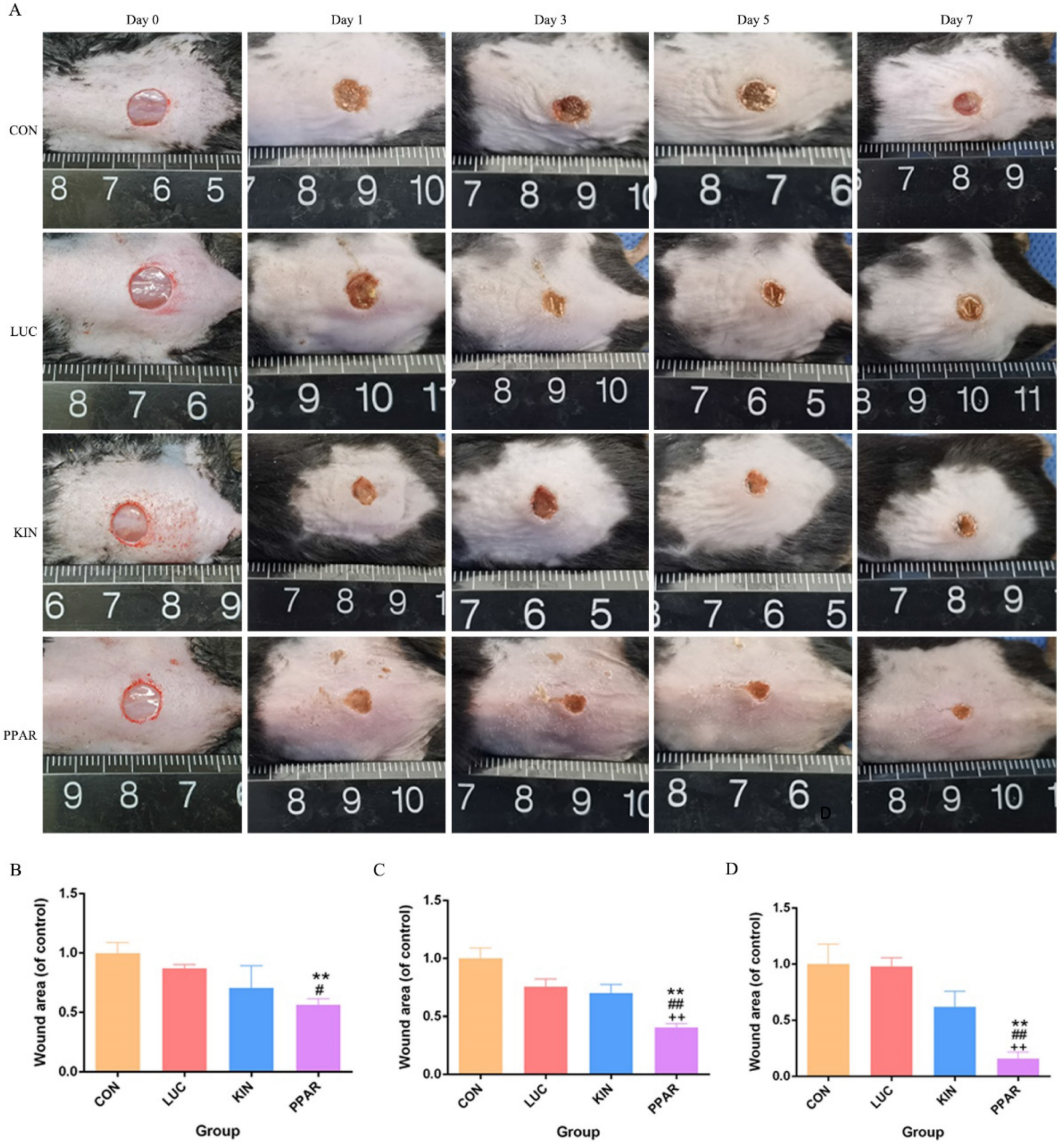
** Therapeutic impact of PPARγ-modRNA in a murine full-thickness skin defect model.** Gross observations and photographic documentation post-surgery in the CON, LUC, KIN, and PPAR groups at 0, 1, 3, 5, and 7 days (A). Quantification of wound area on days 3 (B), 5 (C), and 7 (D). Statistical significance is indicated by *p < 0.05, **p < 0.01, ***p < 0.001, vs CON; #p < 0.05, ##p < 0.01, ###p < 0.001, vs LUC; +p < 0.05, ++p < 0.01, ++p < 0.001, vs KIN.

**Figure 4:**
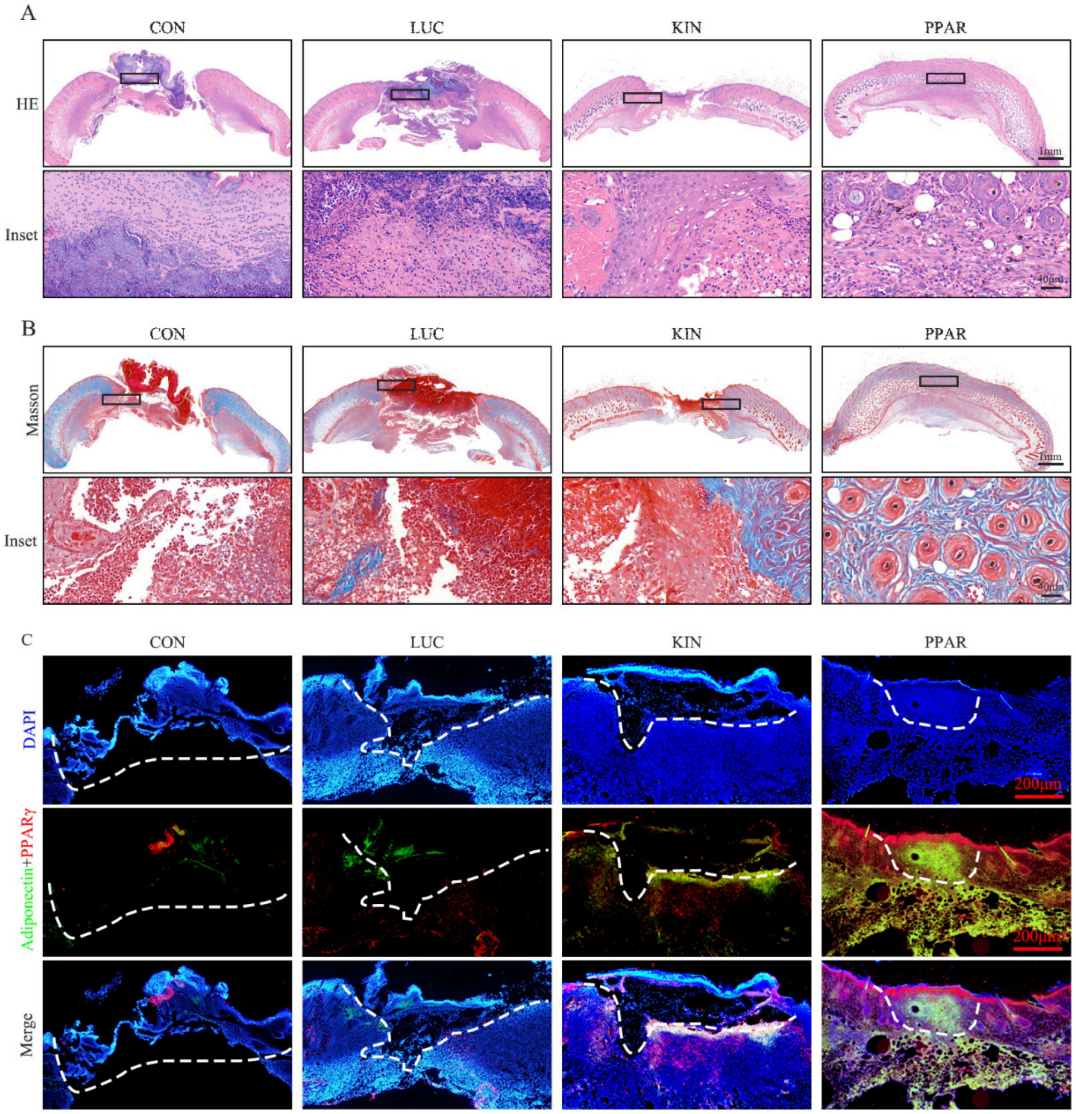
** Histological features of wounds on day 7.** HE staining illustrates increased infiltration of inflammatory cells in the CON and LUC groups, while wounds in the PPAR group exhibit advanced healing with numerous lipid droplets (A). Masson staining showcases reduced and disordered fibrous tissue generation in the CON and LUC groups, contrasting with the PPAR group where fibers appear thicker and more organized compared to the KIN group (B). Immunofluorescence results demonstrate heightened fluorescence intensity for adiponectin and PPARγ in the wounds of the PPAR group, emphasizing enhanced adipogenic differentiation (C).

**Figure 5:**
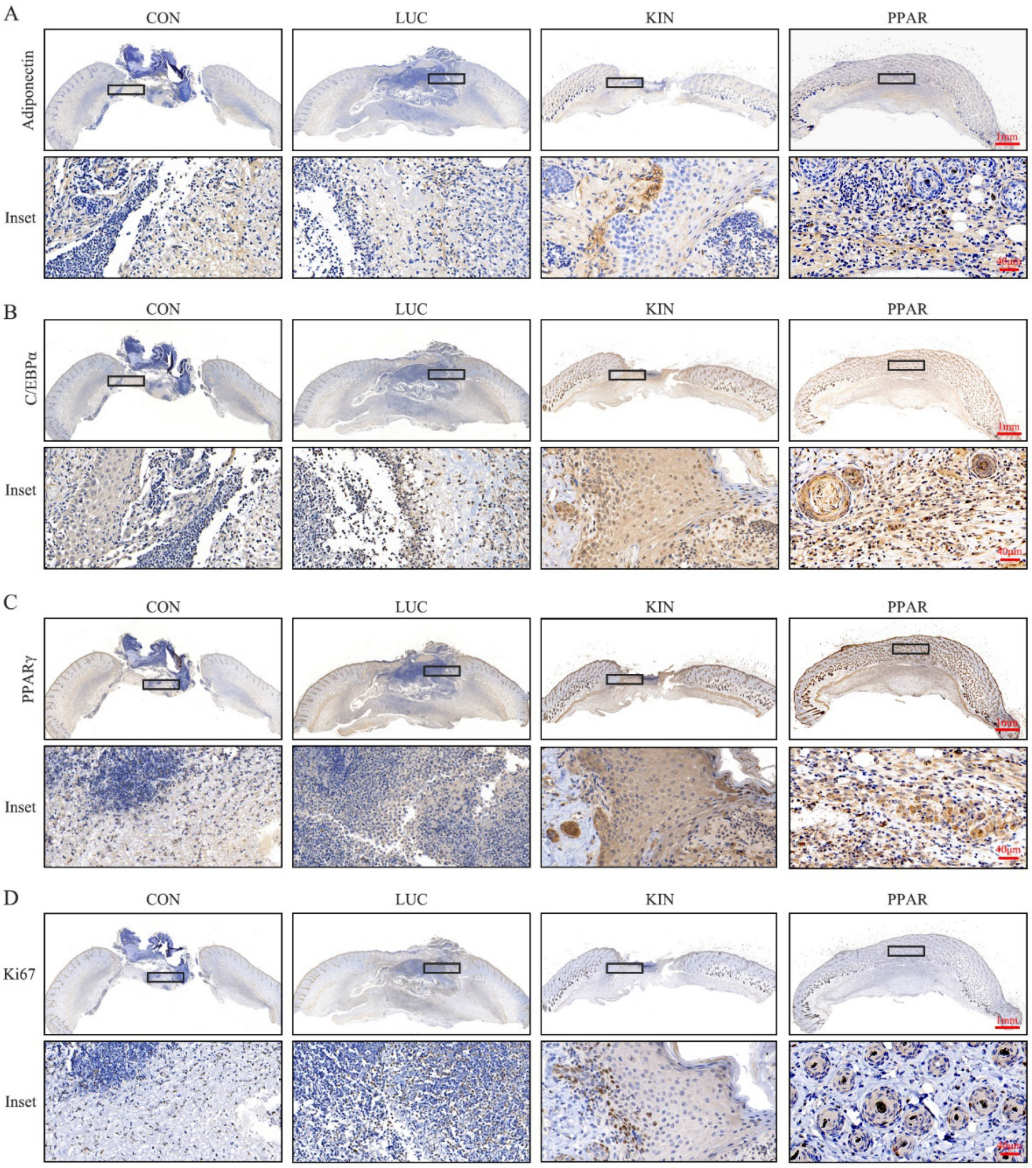
** Molecular markers expression in wounds on day 7.** Immunohistochemistry reveals increased positive expression of adiponectin (A), C/EBPα (B), and PPARγ (C) in the wounds of the PPAR group, followed by the KIN group, consistent with Ki67 results (D).

To assess the potential therapeutic application of PPARγ-modRNA in wound healing, we established a full-thickness skin defect model in mice. Postoperative observations and photographic documentation revealed a notable reduction in wound area on day 3, 5, and 7 in the PPAR group compared to the other three groups, indicating improved and accelerated healing (Fig. 3A-D). Further analysis of wounds on day 7 showed distinct histological features. HE staining indicated increased infiltration of inflammatory cells in the CON and LUC groups, while the wounds in the PPAR group exhibited advanced healing with the presence of numerous lipid droplets (Fig. 4A). Masson staining illustrated reduced and disordered fibrous tissue generation in the CON and LUC groups, contrasting with the PPAR group where fibers appeared thicker and more organized compared to the KIN group (Fig. 4B). Immunofluorescence results demonstrated heightened fluorescence intensity for adiponectin and PPARγ in the wounds of the PPAR group (Fig. 4C). Immunohistochemistry further revealed increased positive expression of adiponectin, C/EBPα, and PPARγ in the wounds of the PPAR group, followed by the KIN group, consistent with the Ki67 results (Fig. 5A-D). In summary, the application of PPARγ-modRNA in wound healing demonstrated significant improvements, as evidenced by enhanced macroscopic wound closure, histological features, and molecular markers associated with adipogenesis and tissue regeneration.

### Transcriptomic profiling reveals PPARγ-modRNA induced differential gene expression patterns

**Figure 6:**
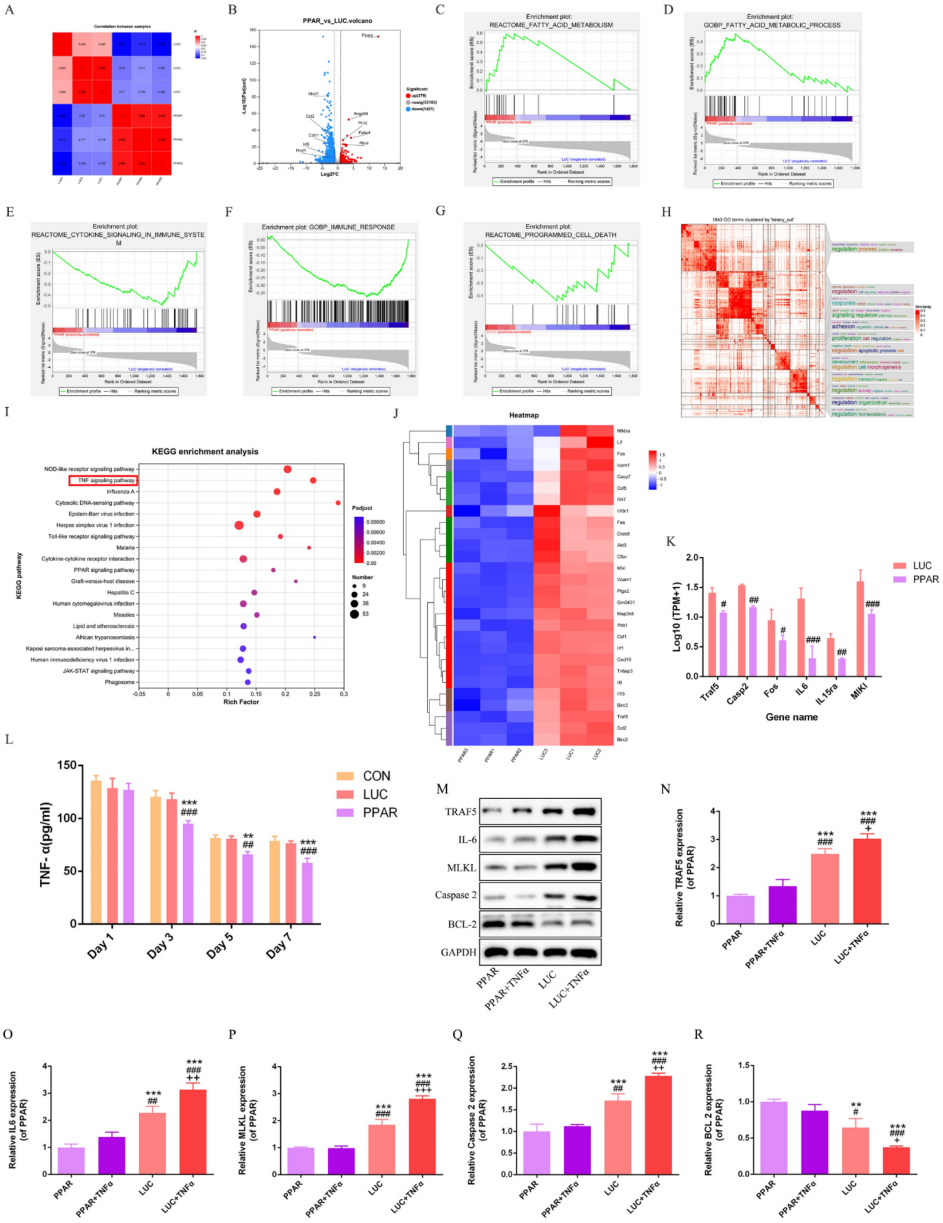
** Transcriptomic profiling and TNF signaling pathway analysis.** RNA-seq analysis reveals a significant correlation and shared biological profile at the gene expression level between the LUC and PPAR groups (A). 379 genes upregulated and 1421 genes downregulated in the PPAR group compared to LUC (B). Enrichment analysis highlights peaks in fatty acid-related pathways, reduced immune response, and decreased cell death in the PPAR group (C-G). Binary clustering and SimplifyEnrichment analysis of 1843 Gene Ontology terms emphasize significant enrichment in cell regulation, proliferation, and apoptosis (H). Reactome enrichment analysis identifies a predominant association with the TNF signaling pathway (I). A comparative heatmap analysis shows differential expression of TNF signaling pathway genes, including suppressed expression of TRAF5, IL-6, MLKL, and Caspase-2 in the PPAR group (J-K). ELISA demonstrates a significant reduction in TNF-α levels in the PPAR group on postoperative days 3, 5, and 7 compared to CON and LUC groups *in vivo* (L). *In vitro* analysis post TNF-α treatment reveals increased expression of TRAF5, IL-6, MLKL, and Caspase-2, indicating TNF signaling pathway activation. PPARγ-modRNA reverses this trend, enhancing BCL-2 expression in the PPAR group (M-R). For panel K, statistical significance is indicated by: #p < 0.05, ##p < 0.01, ###p < 0.001, vs LUC. For panel L, statistical significance is indicated by: *p < 0.05, **p < 0.01, ***p < 0.001, vs CON; #p < 0.05, ##p < 0.01, ###p < 0.001, vs LUC. For panels N-P, statistical significance is indicated by: *p < 0.05, **p < 0.01, ***p < 0.001, vs PPAR; #p < 0.05, ##p < 0.01, ###p < 0.001, vs PPAR+TNFα; +p < 0.05, ++p < 0.01, +++p < 0.001, vs LUC.

RNA-seq analysis of the LUC and PPAR groups unraveled distinct gene expression signatures, with a significant correlation indicating a shared biological profile at the gene expression level (Fig. 6A).

A total of 379 genes were upregulated and 1421 genes downregulated in the PPAR group compared to LUC (Fig. 6B). Enrichment analysis highlighted associations between differentially expressed genes and specific biological pathways, showing notable peaks in fatty acid-related pathways, reduced immune response, and decreased cell death in the PPAR group compared to the LUC group (Fig. 6C-G). Binary clustering and simplifyEnrichment analysis of 1843 Gene Ontology (GO) terms emphasized significant enrichment in terms related to cell regulation, proliferation, and apoptosis (Fig. 6H). Reactome enrichment analysis identified a predominant association with the TNF signaling pathway (Fig. 6I). A comparative heatmap analysis between the LUC and PPAR groups demonstrated the differential expression of genes associated with the TNF signaling pathway, such as TRAF5, IL-6, MLKL, and Caspase-2. Notably, these genes exhibited suppressed expression in the PPAR group (Fig. 6J-K). Further exploration of the TNF signaling pathway's role in wound healing involved additional analysis of the CON, LUC, and PPAR groups. ELISA results demonstrated a significant reduction in TNF-α levels in the PPAR group on postoperative days 3, 5, and 7 compared to the CON and LUC groups *in vivo* (Fig. 6L). *In vitro* cell analysis post TNF-α treatment revealed increased expression of TRAF5, IL-6, MLKL, and Caspase-2, indicative of TNF signaling pathway activation. In particular, this trend was reversed by PPARγ-modRNA, with enhanced BCL-2 expression in the PPAR group (Fig. 6M-R). These results offer a holistic understanding of the transcriptomic changes prompted by PPARγ-modRNA, emphasizing its capacity to potentially facilitate wound healing mechanisms. Notably, this modulation appears to involve the simultaneous promotion of adipogenic differentiation and the suppression of inflammatory responses during the intermediate to late stages of wound healing.

## Discussion

Wound healing represents a dynamic interplay of cellular and molecular events orchestrated to restore tissue integrity. Our study explores the therapeutic potential of PPARγ-modRNA in wound healing, aiming to deepen our understanding of the underlying mechanisms and introduce innovative approaches to regenerative medicine. In the context of wound healing, the delicate balance between inflammation, cell proliferation, and tissue remodeling is crucial for successful repair. PPARγ, known for its roles in adipogenic differentiation and anti-inflammatory responses, served as a focal point for our investigation^24^. Using chemically modified mRNA to overexpress PPARγ, our study introduces a novel dimension by achieving sustained nuclear entry. This groundbreaking achievement expands the toolkit for targeted nuclear modulation, offering far-reaching applications not only in wound healing but also across diverse biological contexts.

In our exploration, the dynamic temporal profile of PPARγ expression post-transfection in 3T3-L1 preadipocytes revealed a nuanced pattern over 24 hours, showcasing the successful nuclear overexpression of PPARγ. This innovative departure from traditional modRNA applications opens a new dimension for precise nuclear modulation. Transcriptomic analysis further provided a detailed molecular narrative, shedding light on the upregulation of genes associated with fatty acid metabolism. Simultaneously, a concurrent downregulation of immune response and cell death pathways was observed, highlighting the crucial role of PPARγ-modRNA in orchestrating molecular changes conducive to effective wound repair. One intriguing observation was the suppression of genes within the TNF signaling pathway by PPARγ-modRNA. This suggests a potential anti-inflammatory mechanism orchestrated by PPARγ, offering a unique perspective on the interplay between PPARγ and inflammatory signaling pathways. Consistent with previous research findings, which have demonstrated the anti-inflammatory effects of PPARγ, our study provides a novel dimension to the understanding of this mechanism. The timely modulation of the TNF signaling pathway is crucial for effective wound healing, as early inflammation is pivotal for initial stages of wound closure^25^. However, prolonged inflammation can impede optimal healing. The administration of PPARγ-modRNA, with its demonstrated anti-inflammatory effects, aligns with the requirement for timely anti-inflammatory responses in the mid to late stages of wound repair, ensuring a balanced and favorable environment for optimal regeneration. To explore deeper into the relationship between TNF signaling and PPARγ, it is essential to consider the intricate crosstalk between inflammatory pathways and PPARγ-mediated processes. The suppression of TNF signaling by PPARγ-modRNA may represent a fine-tuned regulatory mechanism, where PPARγ acts as a master orchestrator to balance inflammatory responses and promote a regenerative microenvironment. The translational leap from cellular models to a murine full-thickness skin defect model enhanced the relevance of our findings. Accelerated wound closure in the PPAR group underscored the therapeutic potential of PPARγ-modRNA, emphasizing its efficacy *in vivo*. Histological examinations further unveiled a regenerative microenvironment characterized by reduced inflammation and heightened adipogenic differentiation.

**Advantages of PPARγ-modRNA:** In comparison to other gain-of-function methods, PPARγ-modRNA presents several notable advantages:

**Transient Expression:** One of the primary benefits of using modRNA is its transient expression profile. Unlike DNA-based methods, which can result in stable and potentially permanent genetic modifications, modRNA allows for controlled, temporary expression of the target gene. This transient nature reduces the risk of insertional mutagenesis and unwanted long-term effects, making it a safer option for therapeutic applications^26^.

**Reduced Risk of Genomic Integration:** DNA-based methods, particularly those involving viral vectors, carry a risk of random genomic integration, which can lead to insertional mutagenesis and disrupt normal gene function. ModRNA, on the other hand, does not integrate into the host genome, thus eliminating this risk and enhancing the safety profile of the therapy^27^.

**Rapid and Efficient Protein Expression:** ModRNA can be quickly translated into protein upon delivery into cells, ensuring rapid onset of the desired therapeutic effects. This is particularly advantageous in acute settings such as wound healing, where timely intervention is crucial^28^.

**Immunogenicity and Stability:** Advances in mRNA technology have significantly reduced the immunogenicity of synthetic mRNA. Chemical modifications and optimized formulations enhance the stability and translation efficiency of modRNA, making it a viable alternative to traditional DNA-based approaches^26^.

**Versatility and Customization:** ModRNA can be easily designed and synthesized to encode any protein of interest, providing a versatile platform for various therapeutic applications. This flexibility allows for the rapid development and testing of new therapies without the need for extensive genetic engineering^29^.

Given these advantages, we chose modRNA over more stable DNA-based methods for this study to leverage its safety, efficiency, and versatility. Our findings support the potential of PPARγ-modRNA as a promising approach for enhancing adipogenesis and promoting wound healing, paving the way for future research and therapeutic development.

While our study offers valuable insights into the potential of PPARγ-modRNA in wound healing, several limitations should be acknowledged to provide a balanced interpretation of the findings. Our study is predominantly focused on unraveling the adipogenic effects induced by PPARγ modulation in the context of wound healing. Although adipogenesis holds pivotal significance in tissue repair, it represents only one facet of the intricate processes integral to wound healing. The exclusive emphasis on adipogenesis might inadvertently overshadow other critical dimensions of wound repair, demanding future investigations that adopt a more comprehensive approach to encompass diverse cell types and signaling pathways. Moreover, while our study showcases the observable outcomes resulting from PPARγ-modRNA treatment, the underlying molecular mechanisms steering these effects remain inadequately characterized. Achieving a deeper mechanistic understanding is crucial, particularly in delineating the intricate pathways facilitating PPARγ-modRNA nuclear entry and subsequent downstream regulatory events. This mechanistic elucidation is indispensable for cultivating a holistic comprehension of PPARγ-modRNA's impact on wound healing, paving the way for more nuanced therapeutic interventions.

In conclusion, our exploration of wound healing unveils the intricate interplay of molecules and cells, presenting a revolutionary approach through PPARγ-modRNA. The integration of molecular insights, innovative methodologies, and *in vivo* validations suggests that PPARγ-modRNA holds promise as a novel approach for enhancing wound healing interventions. The quest for unraveling the full potential of PPARγ-modRNA in wound healing continues, pointing toward a future where regenerative medicine reshapes the clinical treatment landscape.
